# Assessment of contextual factors shaping delivery and uptake of isoniazid preventive therapy among people living with HIV in Dar es salaam, Tanzania

**DOI:** 10.1186/s12879-022-07867-5

**Published:** 2022-11-24

**Authors:** Renatus Fabiano Nyarubamba, Adam Silumbwe, Choolwe Jacobs, Patricia Maritim, Paschal Mdoe, Joseph Mumba Zulu

**Affiliations:** 1 Department of Health Policy and Management, School of Public Health, University of Zambia, Ridgeway Campus, Lusaka, Zambia; 2 Department of Epidemiology and Global Health, Umeå University, 901 87 Umeå, Sweden; 3 Department of Epidemiology and Biostatistics, School of Public Health, University of Zambia, Ridgeway Campus, Lusaka, Zambia; 4 Haydom Lutheran Hospital, Manyara, Tanzania; 5 Division of Nursing, Midwifery and Social Works, University of Manchester, Faculty of Biology Medicine and Health, Manchester, UK; 6 Ministry of Health, Community Development, Gender Elderly and Children, Dodoma, Tanzania

**Keywords:** Isoniazid preventive therapy, Tuberculosis, Delivery, Uptake, HIV, Tanzania

## Abstract

**Background:**

Tuberculosis has remained a leading cause of death among people living with HIV (PLHIV) globally. Isoniazid preventive therapy (IPT) is the recommended strategy by the World Health Organization to prevent TB disease and related deaths among PLHIV. However, delivery and uptake of IPT has remained suboptimal particularly in countries where HIV and TB are endemic such as Tanzania. This study sought to assess contextual factors that shape delivery and uptake of IPT in Dar es Salaam region, Tanzania.

**Methodology:**

We employed a qualitative case study design comprising of in-depth interviews with people living with HIV (n = 17), as well as key informant interviews with clinicians (n = 7) and health administrators (n = 7). We used thematic data analysis approach and reporting of the results was guided by the Consolidated Framework for Implementation Research (CFIR).

**Results:**

Characteristics of IPT such as aligning the therapy to individual patient schedules and its relatively low cost facilitated its delivery and uptake. On the contrary, perceived adverse side effects negatively affected the delivery and uptake of IPT. Characteristics of individuals delivering the therapy including their knowledge, good attitudes, and commitment to meeting set targets facilitated the delivery and uptake of IPT. The process of IPT delivery comprised collective planning and collaboration among various facilities which facilitated its delivery and uptake. Organisational characteristics including communication among units and supportive leadership facilitated the delivery and uptake of IPT. External system factors including HIV stigma, negative cultural and religious values, limited funding as well as shortage of skilled healthcare workers presented as barriers to the delivery and uptake of IPT.

**Conclusion:**

The factors influencing the delivery and uptake of IPT among people living with HIV are multifaceted and exist at different levels of the health system. Therefore, it is imperative that IPT program implementers and policy makers adopt multilevel approaches that address the identified barriers and leverage the facilitators in delivery and uptake of IPT at both community and health system levels.

**Supplementary Information:**

The online version contains supplementary material available at 10.1186/s12879-022-07867-5.

## Background

Tuberculosis (TB) and HIV co-infection remain a major global public health problem, particularly in low-and-middle income countries (LMICs) [1]. A total of 1.5 million people died from TB in 2020 and10 million fell ill, with the largest number of new cases occurring in South-East Asia and Africa, respectively [2]. HIV/AIDS has significantly fuelled TB incidence as evidence shows that people living with HIV (PLWHIV) have 18 times higher likelihood of developing TB disease compared to those who are HIV negative [3]. TB is the most common opportunistic infection and the major cause of deaths in PLWHIV [1]. In 2020, about 215 000 people died of HIV-associated TB [3].

The World Health Organisation (WHO) recommends the use of isoniazid preventive therapy (IPT) as a global public health strategy to reduce  TB incidences among PLWHIV [4]. IPT is an evidence-based intervention that entails provision of isoniazid, one of the most effective bactericidal, anti-TB drugs, for a duration of 6–12 months [5]. According to the WHO, IPT reduces the risk of TB by more than 32% among PLWHIV [6]. Although antiretroviral therapy (ART) is a significant intervention to treat TB in PLWHIV, HIV positive patients receiving ART remain susceptible to active TB disease [7]. Besides early ART initiation, IPT is a key intervention to prevent TB among PLWHIV [8]. Many countries where TB and HIV are co-endemic have scaled up delivery of IPT to PLWHIV [1].

Despite the effectiveness of IPT in preventing progression of latent TB infection into active TB disease, the delivery and uptake of IPT among PLWHIV has remained sub-optimal in most countries [9, 10]. For example, in the African region, South Africa had majority of newly diagnosed PLWHIV put on IPT in 2019 at 69% and Eswatini had the lowest at about 1% [2]. Further, in Ethiopia and Zambia, IPT implementation has faced several challenges including poor patient adherence, fear of side effects, development of isoniazid resistant TB, and lack of commitment by health managers to scale up the program [11, 12].

In Tanzania, an estimated 1.7 million people were living with HIV in 2019 [13]. Of these, 40,000 contracted TB and 40% died [14]. The incidence rate of TB among PLWHIV in Tanzania is as high as 16.7 cases per 1000 person-years [15]. However, only about 59% of PLWHIV eligible for IPT have been put on treatment which is lower than the WHO 90% target [14].

Tanzania adopted IPT in 2010 and scaled it up across the health system in 2011 [16]. Although there has been progress in delivering IPT to PLWHIV in Tanzania, challenges remain. For example, only about 58% six-month IPT completion was reported among PLWHIV attending care and treatment in Dar es Salaam between 2013 and 2017 [17]. Further, a retrospective cohort study found IPT initiation of 14.38% between 2012 and 2016 which is too low to achieve the global treatment targets [18].

There are various studies that have looked at factors affecting implementation of IPT in other countries [12, 19, 20]. However, in Tanzania, studies have primarily focused on determining the effectiveness of IPT, completion rate of IPT regimen and knowledge on IPT [21–23]. There has been limited assessment of the contextual factors that shape delivery and uptake of the IPT intervention. This study aimed to bridge this gap through assessing contextual factors that shape delivery and uptake of the IPT intervention in Dar es Salaam, Tanzania using the consolidated framework for implementation research so as to inform policy guidelines towards effective implementation of IPT [24].

## Methods

### Theoretical framework

We used the consolidated framework for implementation research (CFIR) to assess the context in which IPT is delivered and how it shapes IPT uptake among PLHIV [25]. The CFIR was developed to guide systematic assessment of multilevel contexts that influence implementation of an intervention. CFIR is a determinant framework consisting five domains that we used to categorise the contextual factors that shape delivery and uptake of IPT [26]. These domains include the characteristics of the intervention, inner setting, outer setting and individuals involved and process of implementation [25]. We used the CFIR to guide data collection, data analysis, coding, and interpretation of the findings.

### Study design

We used an exploratory qualitative case study design [27, 28]. The case comprised the IPT program including the delivery systems, related health facilities, health services managers, health providers and PLWHIV in Dar es Salaam region, Tanzania. This design is most appropriate because it allowed us to explore in detail the IPT intervention and its interaction with the contextual factors that shape its delivery and uptake [28].

### Study setting

This study was conducted in Dar es Salaam, a major commercial city and key contributor of new TB cases in Tanzania, with 20% of all TB cases in the country notified from this region in 2017 [29]. The National HIV impact survey reported a regional HIV prevalence of 4.7% among adults 15 years and older [30]. Dar es Salaam is the most populous city in Tanzania with a population of about 5.5 million [31]. In relation to TB/HIV services, in 2018 Dar es Salaam had a total of 123 HIV clinics where IPT is offered [30].

### Study participants

The study participants comprised of 7 health administrators at different levels of IPT implementation from the government and non–governmental organizations, 9 clinicians and 17 people living with HIV. All participants were at least 18 years old. Table 1 shows the different categories of participants who took part in the study.

**Table 1:** Categories of participants

S/No.	Participant category	Number of individuals interviewed
Key informant interviews		
1	Health administrators	7
2	Clinicians (nurses and doctors)	9
	Sub total	16
In-depth interviews		
3	Patients/PLHIV	17
	Sub total	17
Total		33

### Sampling and recruitment of study participants

The study utilized purposive sampling methods to select participants and achieve a maximum-diversity sample. Health administrators and the providers were selected based on their experience in the delivery of IPT services whilst the PLWHIV were supposed to either be on or had completed the IPT regimen. Those who did not meet these criteria were excluded. The clinics were purposively sampled based on their performance in IPT delivery in consultation with the district health administration. Two clinics were selected from each of the five districts within Dar es Salaam including Kigamboni, Temeke, Ilala, Ubungo and Kinondoni.

The PLWHIV were recruited at the clinics with assistance from the TB/HIV focal person and sister in-charge who worked with the researcher to identify patients who were eligible among those who attended the clinic on the interview day. However, the researcher had the final choice of which patient to be interviewed from among the ones recommended. Two patients were interviewed from each of the clinics. We tried to balance the sex and general representation of various social categories in the way the study participants, particularly PLWHIV were recruited.

### Data collection

The data collection comprised of key informant and in-depth interviews. Having obtained permission to collect data from all the districts and selected clinics, patient files were reviewed to confirm the IPT regimen. The interviews were face-to-face except for one done by phone. The interviews were conducted in a private room using an interview guide (Additional file 1) and lasted between 30 and 60 min. The interview guide was developed based on CFIR framework [25].

Due to the COVID-19 pandemic, a distance of at least 2m between interviewer and the interviewee was observed. Moreover, both the interviewers and the study participants wore protective masks. Each interview was audio recorded and the interviewer took some notes during the sessions. All interviews were conducted by the first author RFN, a postgraduate  public health student and Tanzanian male doctor with training in qualitative methods in implementation research at the University of Zambia. Data were collected between January and February 2021.

Although we achieved saturation of data with 25 interviews, we continued to a total of 33 which, however, did not reveal new information (Table 1). The concept of “theoretical saturation,” a point when additional data does not produce new information, is an accepted standard for determining the sample of a qualitative inquiry [32]. In total, we conducted 16 key informant interviews and 17 in-depth interviews (Table 1).

### Data analysis

This study employed thematic analysis, an approach in which themes, patterns and relationships are identified, analyzed and presented from the qualitative data [33]. Recorded interviews were transcribed verbatim in Swahili language. Thereafter, English version of the transcripts were developed to enable detailed review by a research team. The transcripts were then read several times to develop codes from which themes were synthesized. Codes and themes were developed both deductively using the CFIR and inductively using the context specific emergent issues from the data (Table 2) [25]. All the team members iteratively discussed the final coding framework and agreed on the relevant structure of presenting the coding reports. Each member manually coded a specific number of transcripts and discussed with the research team to reach consensus on emergent issues.

**Table 2:** CIFR domains and the emergent themes

CFIR Domain	CFIR construct	Key themes	Barrier/facilitator
Characteristics of intervention	Source of intervention Implementation costs	Proven effectiveness of IPT in preventing active TB Adaptability of IPT to patient schedules IPT provided for free of charge Adverse side effects of IPT Indirect costs incurred by patients IPT pills burden	Facilitator Facilitator Facilitator Barrier Barrier Barrier
Characteristics of individuals	Knowledge and belief about the intervention	Good knowledge and attitude among clinicians and patients Accepting HIV status and having taken IPT before	Facilitator Facilitator
Process of implementation	Engaging	Collective planning prior to IPT delivery Key stakeholder engagement Collective evaluation of IPT delivery and uptake	Facilitator Facilitator Enabler
Inner settings	Organizational culture Implementation climate	Communication among staffs participating in IPT delivery Commitment of institutional leadership Shortage of staff and lack of diagnostics facilities	Facilitator Facilitator Barrier
Outer settings	Cosmopolitanism Sociocultural	Network of health facilities sharing resources HIV related stigma Some Negative cultural and religious values Poverty and weak health systems	Enabler Barrier Barrier Barrier

### Ethical considerations

This study obtained ethical approval from the University of Zambia Biomedical Research Ethics Committee (UNZABREC) (REF. 1022-2020) to conduct the research. Approval was also provided by the National Institute of Medical research in Dar es Salaam, Tanzania (NIMR) (Ref: NIMR/HQ/R.8a/Vol.IX/3541) where the data was collected. All prerequisite authorisations were obtained from the Tanzania Ministry of Health. We also obtained permission from the regional and district office, and the hospital management before data collection. All participants (> 18 years) provided written, informed consent to participate in the study. If participants were not literate, a witness was required to be present during the consenting process and sign consent on their behalf. The participants gave separate consent to being audio recorded. Confidentiality was assured by maintaining anonymity of participants who were assigned a unique codes to de-identify them during data reporting.

## Results

Factors that shape delivery and uptake of IPT were identified. The contextual factors were assessed at various levels and are presented according to the domains of the CFIR;  characteristics of IPT intervention, characteristics of individuals delivering and taking IPT, organisational process of delivering the IPT intervention, inner settings of the organisations/facilities delivering IPT as well as outer settings affecting IPT delivery and uptake as shown in Table 2. The results presented reflect the similar and diverging views expressed by the study participants with examples of verbatim quotes to illustrate the identified themes.

### Characteristics of the IPT intervention

*Source of intervention* Although IPT was adopted based on recommendation of the World Health Organisation, observed evidence of its effectiveness in preventing active TB cases among PLWHIV and aligning of the therapy to individual patient schedules led to it being supported by local actors such as healthcare providers, the media, government, civil society organizations and patients.

“*We got guidelines from WHO that eligible (HIV) clients should be started on IPT. Since we do follow and contextualize WHO directives, we started processes of implementing them. Despite being sourced outside, I don’t think this was of any problem because we as government and other stakeholders worked together on this like many other interventions”* **(KII, Health Administrator)**

*Implementation cost* IPT is provided free of charge at all the health facilities like other TB and HIV related services, which facilitated its delivery and uptake. However, the costs of transportation and clinical investigations such as chest X-ray posed a barrier to delivery and uptake of IPT. Further, adverse side effects and complexities associated with the use of IPT such as long duration, pill burden, nausea and numbness discouraged some of the patients from completing the IPT regimen.

*“First of all, we take the drugs for long time, six months is quite long. So, if possible, the duration perhaps should be reduced. Yes, you take once daily but for six months. It’s too long. There are some people who can’t take for all six months and thus stop taking the drugs”* **(IDI, Patient PLWHIV Client)**

### Characteristics of individuals involved in IPT delivery and uptake

*Knowledge and beliefs about the intervention* The health workers demonstrated good understanding of IPT. They explained that it prevents the progress of latent TB infection to active TB disease. This understanding helped them to build commitment towards delivery of the IPT intervention. They stated that delivering IPT was critical to reduce TB related deaths among PLWHIV. Additionally, the health workers indicated that confidence in the ability to deliver IPT as per guidelines was another important factor facilitating delivery and uptake of IPT. One clinician had this to say.

*“IPT is a critical intervention to protect people living with HIV from getting active TB disease. We provide it to all eligible clients regardless of their age for a duration of six month. This medication is not complicated and its one of those that we can easily administer to eligible patients”* **(KII, Clinician)**

On the contrary, some PLWHIV could neither explain what IPT is nor why they were on the regimen. When asked about how she understood IPT, one patient replied:

*“I don’t know what it means. We are just given drugs for tuberculosis, but I can't really explain what IPT means or how it works in our bodies”* **(IDI, Patient, PLWHIV)**

Acknowledging one’s HIV status was important in determining whether a patient could accept HIV related interventions such as IPT. Patients with at least three months on the IPT regimen were enthusiastic to continue taking the intervention to completion. Such patients considered IPT as part of their lives compared to recent initiators who may be struggling to get used. One patient opined that.

*I have been on this treatment for the past three or more moths. I use it (Isoniazid) just like I am using Panadol…... Even if am going out, I still remember that I should take the drug first* **(IDI, Patient, PLWHIV)**

### Processes of delivering IPT intervention

*Engaging* Planning for delivery of IPT services was reported to be collectively done. Various stakeholders including those at the lowest level of the health system were engaged during the different phases of IPT implementation and decision making. These activities promoted stakeholder ownership of the IPT intervention hence facilitating its delivery and uptake.

*“Since I joined HIV services, I think one of the good things I have seen is that if there are changes, we clinicians are usually informed, and our leaders will go to the meetings to plan how to adopt the changes in our works. I like that because it makes my work easy”* **(KII, Clinician)**

*“There are monthly reports that indicate how many patients we have been able to cover and how many patients have completed the regimen within a particular month. So, every month we get a report on IPT completion”* **(KII, Clinician)**

PLWHIV who were adherent and had been on the IPT regimen for a long time were engaged as treatment champions to assist with delivery of the intervention as peer-to-peer counsellors. Use of champions to deliver IPT created trust in the intervention hence facilitating its uptake among PLWHIV. This was supported by clinicians who intimated that.

*Speaking of champions…patients trust them a lot. They regard them (champions/expert clients) as facility workers. They agree with them* **(KII, Clinician)**

### Characteristics of institutions delivering IPT intervention

*Organisational culture* Good working relationships and communication among units within the health facilities delivering TB/HIV services such as laboratory, radiology, and pharmacy among others helped to ensure uninterrupted and coordinated delivery of the IPT intervention. One clinician attested to this by stating.

*“(….) every section does its job: laboratory, radiology unit and counselling. All people involved play their roles to help patients. For me, I see a patient, listen to him, and prescribe. But when I want blood tests, I send them outside to that room opposite to my office which is our Laboratory”* **(KII, Clinician)**

*Implementation climate* The IPT implementation climate characterised by a learning environment that enables sharing of experiences, skills and delegation of tasks had positive influence on the delivery and uptake of IPT. Leaders and managers of the IPT program at the district and facility levels were reported to be actively engaged in IPT services through mentorship and advocacy. This created a supportive environment for the delivery and uptake of IPT services among PLWHIV as one clinician explained.

*“She (District HIV Coordinator) has a lot to do and meetings to attend. She sometimes cannot do all and she will ask some of us to do it. For example, today she is not here, she went to the other CTC for supervision, so she asked me to prepare this report. (……) Of course, it feels good when you can also teach others since it makes you confident”* **(KII, Clinician)**

However, the inadequate number of clinicians and huge volumes of patients at some of the facilities compromised the delivery and uptake of IPT services. Further, the lack of some diagnostic services meant that patients had to be referred to other facilities to conduct their investigations which negatively affected the delivery and uptake of IPT services.

*“…when you go to facilities with enough staff, there is an exit desk to ensure a client has received all correct services and the next appointment is provided. However, there cases where not all diagnostics services are provided at a particular facility and then you must go elsewhere do the test and come back to see the clinician”* **(KII, Clinician)**

### Factors external to the organizations delivering IPT

*Cosmopolitanism* The facilities delivering IPT in the Dar es Salaam region have developed a network amongst themselves that enables sharing of resources such as IPT drugs when there is a deficit to ameliorate service delivery interruptions. This networking has also resulted in competitive pressure among the facilities towards attaining treatment targets which are collaboratively set with partner organizations in the region.

*“Cooperation between our facility and others exists and may include sharing of drugs. When they have no drugs, they will borrow from us because here drugs are easily available and one may tell you “Give me three boxes, I will bring it back when I receive my stock”* **(KII, Clinician)**

*Sociocultural* Although disclosure of one’s HIV status to relatives such as spouses and parents was generally accepted as important for getting treatment support, the fear of HIV stigma caused some of the patients not to disclose, which affected their uptake IPT services. When asked whether it was important to disclose the HIV status, one patient narrated.

*“Yes, it is very important (disclosing HIV status) because you may get sick, or it may be your clinic visit date and someone should take you. You fail to ask someone to collect your drugs. It is very, very important but the question is how I disclose to someone. That’s a challenge that gives me headache”* **(IDI, Patient)**

To some of the patients, religious teachings were reported to influence their decision to take IPT. For example, some HIV patients refused to take IPT because they thought that they would not contract TB because they believed in God. Describing an incidence that she once experienced, one clinician narrated.

*“(….) we used to tell her to bring back the empty packages for verification, now since she was a religious person, she could not lie. She used to keep the drugs at home and when she felt like we insisted much to see the used empty packages, she brought all the drugs: “here are your drugs, I am not taking it. I am (mentions her religion), I believe I won’t suffer from TB disease”* **(KII, Clinician)**

This observation was also shared by a patient who stated that.

*I was given some small tablets (Isoniazid) to take to prevent Tuberculosis. But our pastor preached that we could get healed by prayers only. So, I stopped using the medicines until later when I fell very sick, and the nurse here told me I should not stop using medicines* (**IDI, Patient**).

## Discussion

Characteristics of IPT such as aligning the therapy to individual patient schedules and its relatively low cost facilitated its delivery and uptake. On the contrary, perceived adverse side effects of IPT negatively affected the delivery and uptake of IPT. Characteristics of individuals delivering the therapy including their knowledge and good attitudes, commitment to meeting set targets facilitated the delivery and uptake of IPT. The process of IPT delivery comprised collective planning and collaboration among various facilities which facilitated its delivery and uptake. Organisational characteristics including communication among units and supportive leadership facilitated the delivery and uptake of IPT. External system factors including HIV stigma, negative cultural and religious values, limited funding as well as shortage of skilled healthcare workers presented as barriers to the delivery and uptake of IPT.

### Characteristics of the IPT intervention shaping delivery and uptake

Characteristics of the IPT intervention may act as barriers to its delivery and uptake. For example, the large number of isoniazid pills to be taken, duration, inconsistent availability of the drug and associated side effects such as neuropathy result in patients shying away from the intervention. Similar findings have been reported in studies conducted Kenya and Ethiopia [9, 11]. These studies also highlight critical health system barriers to the delivery and uptake of IPT such as lack of commitment by health managers to scale up and lack of an integrated monitoring and evaluation systems. On the other hand, good attributes of the IPT intervention such as its adaptability to patient schedules, IPT’s proven effectiveness as well as it being provided free of charge facilitate its delivery and uptake as reported in similar studies [22, 34–36]. However, indirect costs incurred by the PLWHIV to access healthcare pose as a barrier to IPT delivery and uptake.

### Characteristics of the individuals involved in delivery and uptake of IPT

Knowledge, beliefs, and attitudes of healthcare providers and PLWHIV towards IPT were found to be key factors shaping its delivery and uptake. The value of PLWHIV having appropriate knowledge is critical to address misunderstandings of IPT’s preventive role in the absence of TB symptoms [37]. One study found that when PLWHIV share wrong beliefs about IPT at community level its delivery and uptake becomes a challenge [20]. In addition, acceptance of one’s HIV status and having taken IPT for a long period of time facilitates its delivery and uptake as reported in similar studies [22, 38]. A possible explanation is that once PLWHIV accept their status and assume responsibility, adherence to treatment comes naturally.

### The organisational processes shaping delivery and uptake of IPT

The engagement of PLWHIV as IPT champions facilitated its delivery and uptake. The IPT champions performed various tasks such as daily health talks, linking fellow patients to services, and participating in counselling sessions. Our finding reaffirm what has already been reported in other studies whereby using people from the affected community to deliver an intervention facilitates its uptake [9, 39]. The use of champions selected among PLWHIV helps to build and sustain trust in not only the IPT intervention, but also the health system in general [39, 40]. This contributes to ensuring that PLWHIV adhere treatment instructions as prescribed by the responsible healthcare workers. Because of their experience, treatment champions also play an important role in countering and myths that otherwise impede PLWHIV from taking the IPT intervention.

### Internal organisational characteristics shaping delivery and uptake of IPT

Leadership support towards the IPT program through ensuring availability of supplies, providing mentorship and supervision facilitated its delivery and uptake. Evidence of the importance of leadership in influencing IPT delivery and uptake has also been documented in several other studies [11, 19]. These studies have found that the lack of support of the leadership overseeing HIV related care has hindered effective delivery and uptake of IPT services. Leadership support within the organisation provides a critical element for channelling implementation efforts towards IPT program success through providing strategic direction and oversight [41].

### Outer settings influencing delivery and uptake of IPT

Sharing experiences and learning from other clinics implementing the IPT program was found to play a significant role in facilitating its delivery and uptake. Networking among clinics implementing IPT not only facilitates communication but also enables exchange of resources which helps to guarantee continuity of IPT services to PLWHIV. This finding is supported by a similar study from Kenya which reports that a lack of interoperability between clinics hindered delivery of IPT services [9].

We found that HIV societal stigma hinders the delivery and uptake of IPT services. However in a southern African study HIV related stigma was reported to have no effect on IPT uptake [37]. A possible explanation to this difference may be because PLWHIV in South Africa were more confident in their ability to navigate stigma by pretending that their medications were for unrelated conditions. On the contrary, PLWHIV in our study seemed to find it a challenge to navigate the HIV/TB co-infection related stigma when it comes to IPT treatment uptake decisions. Furthermore, negative cultural and religious values also hinder delivery and uptake of IPT services as reported in similar studies [11, 42].

This study had several limitations, firstly the collection of data from an urban area, Dar es Salaam meant that most of the views are likely to represent people of better socioeconomic standing. Secondly the small sample size as well as limited variability within entails that we may not be able to aptly transfer our findings to similar settings. Lastly, we take cognizance of the desirability bias that may occur due to the interviewees wanting to give a positive impression of the IPT program performance in their clinic. However, even with these weaknesses, the research team tried to ensure that quality data were collected. The team comprised of seasoned qualitative researchers with experience in implementation and health systems research. During data analysis, the team ensured that the views were triangulated among the different category of participants to ensure trustworthiness of the findings. Further, the iterative process of analysing the data enhanced the validity of our findings. This study has added knowledge to and reaffirmed what is already known about implementation of IPT which can help health policy makers, clinicians, and patients to improve effectiveness of this intervention.

## Conclusion

There are several factors influencing the delivery and uptake of IPT among people living with HIV that are multifaceted and exist at different levels of the health system. This is further compounded by challenges resulting from the inherent weak and fragile health systems in which HIV/TB services are provided. Therefore, it is imperative that program implementers and policy makers adopt multilevel approaches that address the identified barriers and leverage the facilitators in delivery and uptake of IPT at both community and health system levels.

## Supplementary Information

**Additional file 1.** Interview guide _ English version.

## Data Availability

The dataset during and/or analysed during the current study is available from the corresponding author on reasonable request.

## Abbreviations

AIDS: Acquired immunodeficiency syndrome
ART: Anti-retroviral therapy
CFIR: Consolidated framework for implementation research
CTCs: Care and treatment centres (Clinics)
HIV: Immunodeficiency virus
IDI: In-depth interview
IPT: Isoniazid preventive therapy
KII: Key informant interview
NIMR: National Institute of Medical Research
PLWHIV: People living with HIV
TB: Tuberculosis
UNZABREC: University of Zambia Biomedical Research Ethics Committee
WHO: World Health Organization
