# Supplementary material for: Assessment of contextual factors shaping delivery and uptake of isoniazid preventive therapy among people living with HIV in Dar es salaam, Tanzania
Source: BMC Infect Dis. 2022 Nov 24;22:884. doi: 10.1186/s12879-022-07867-5 (PMC9700944; doi:10.1186/s12879-022-07867-5)
Supplement: Supplementary file 1 — Additional file 1. Interview guide _ English version. [file 12879_2022_7867_MOESM1_ESM.docx]

**ASSESSMENT OF CONTEXTUAL FACTORS SHAPING DELIVERY AND UPTAKE OF ISONIAZID PREVENTIVE THERAPY AMONG PEOPLE LIVING WITH HIV IN DAR ES SALAAM, TANZANIA**

**INTERVIEW GUIDE-ENGLISH VERSION**

| **PART I:**  **RESPONDENT DETAILS** | | | |
| --- | --- | --- | --- |
| **Participant ID** | | |  |
| 1 |  | Date of interview |  |
| 2 |  | Place |  |
| Respondent’s: | | | |
| 3 |  | Sex |  |
| 4 |  | Age |  |
| 5 |  | Marital status |  |
| 6 |  | Title/occupation |  |
| **PART II**  **CHARACTERISTICS OF AN INTERVENTION** | | | |
| 7 | Where do you think the IPT intervention came from? | | |
| 8 | How does that affect your view/implementation of the intervention in here? | | |
| 9 | Do you think the intervention needs some changes? What changes? | | |
| 10 | What kind of information or evidence do you know that the intervention is working? | | |
| 11 | What do you think encourages other staff to implement the intervention? | | |
| 12 | How do you think of the complexity of the intervention? | | |
| 13 | What supportive materials do you have that help you implementing the intervention? | | |
| 14 | What is your perception on the quality of the materials, packaging of the intervention? | | |
| 15 | How does that affect implementation of the intervention in your settings? | | |
| **PART III**  **INNER SETTING** | | | |
| 16 | Tell us about your relationship with your colleagues and leaders at work and outside working environment | | |
| 17 | Can you describe the relationship between your organization with influential stakeholders? | | |
| 18 | Do you meet regularly with other staff? | | |
| 19 | How do you get to know about new initiatives, new staff? How did you get to know about IPT? | | |
| 20 | How would you describe the culture of your organization? Of your own setting or unit? | | |
| 21 | How do you think your organization's culture (general beliefs, values, assumptions that people embrace) affect the implementation of the IPT intervention? | | |
| 22 | To what extent are new ideas embraced and used to make improvements in your  organization? | | |
| 23 | Comment on the acceptability of IPT intervention in your organization? | | |
| 24 | How do people feel about current programs/practices/process that are available related to the intervention? | | |
| 25 | How well does the IPT intervention fit with your values and norms and the values and norms within the organization? | | |
| 26 | How well does the IPT intervention fit with existing work processes and practices in your setting? | | |
| 27 | Can you describe how the IPT intervention has been integrated into current processes? | | |
| 28 | What level of involvement has leadership at your organization had so far with the IPT intervention? | | |
| 29 | Do you have sufficient resources to implement and administer the IPT intervention? | | |
| 30 | How do you get the necessary resources (including the drug) to implement the IPT intervention? | | |
| 31 | What kind of training you do receive regarding IPT? For colleagues? | | |
| 32 | What kinds of information and materials about the intervention have already been made available to you? | | |
| 33 | Who do you ask if you have questions about the intervention or its implementation? | | |
| **PART IV**  **OUTER SETTING** | | | |
| 34 | To what extent is your organization aware of the IPT needs of your clients? | | |
| 35 | How far do you think IPT intervention meet the need and expectation of your clients? | | |
| 36 | What barriers your client face in accessing IPT services? | | |
| 37 | How do you get to understand the experience of your clients on IPT intervention? | | |
| 38 | How does that affect their decision to uptake IPT? | | |
| 39 | To what extent do you network with colleagues or people in similar professions/positions outside your setting? | | |
| 40 | What kind of information exchange do you have with others outside your setting, either related to the IPT intervention, or more generally about your profession? | | |
| 41 | To what extent does your organization encourage you to network with colleagues outside your own setting? | | |
| 42 | Can you tell us what you know about any other organizations that have implemented the IPT intervention or other similar programs? | | |
| 43 | To what extent are other organizations implementing the intervention? | | |
| 44 | To what extent are other units within your organization implementing the intervention? | | |
| 45 | To what extent would implementing the intervention provide an advantage for your organization compared to other organizations in your area? | | |
| 46 | What kind of local, state, or national performance measures, policies, regulations, or guidelines influenced the decision to implement the intervention? | | |
| 47 | What kind of financial or other incentives influenced the decision to implement the intervention? | | |
| 48 | What are socio-economic factors that are influencing implementation/use of the IPT intervention? | | |
| **PART V**  **CHARACTERISTICS OF INDIVIDUALS** | | | |
| 49 | Tell us about your understanding of the IPT intervention and how is it implemented | | |
| 50 | What stage of the intervention are you involved in. | | |
| 51 | How do you feel about the IPT intervention in terms of effectiveness, and it being implemented in this facility? | | |
| 52 | How confident are you that you are able to successfully implement the IPT intervention? / How confident are you that you are able to use the intervention? | | |
| 53 | How confident do you think your colleagues feel about implementing/using the intervention? | | |
| 54 | How does your identification with the facility affect implementation of the IPT services? | | |
| 55 | How does motivation, values, competence, capacity, and learning style affect your ability to implement/use the intervention? | | |
| **PART VI**  **PROCESS** | | | |
| 56 | How are you engaging other stakeholders in the implementation of IPT intervention on education, training, other activities? | | |
| 57 | How are you using influential people? | | |
| 58 | Can you comment on the availability and use of formally appointed internal implementation leaders? | | |
| 59 | How is the use of champions in implementing the IPT intervention? How people view these champions? | | |
| 60 | How do you communicate the IPT intervention to your clients? What is the attitude of your clients to IPT services? | | |
| 61 | How far is the intervention carried out as it was planned? | | |
| 62 | How do you measure that implementation of the intervention is effective? | | |
| 61 | Where do you get these feedback (client, colleagues, senior, out of your facility)? | | |

***THANK YOU FOR TAKING PART IN THIS STUDY***

**ASSESSMENT OF CONTEXTUAL FACTORS SHAPING DELIVERY AND UPTAKE OF ISONIAZID PREVENTIVE THERAPY AMONG PEOPLE LIVING WITH HIV IN DAR ES SALAAM, TANZANIA**

**INTERVIEW GUIDE-SWAHILI VERSION**

***Muongozo wa Usahili***

| **SEHEMU YA I:**  **TAARIFA ZA MSHIRIKI** | | | |
| --- | --- | --- | --- |
| Utambulisho | | |  |
| 1 |  | Tarehe ya usahili |  |
| 2 |  | Mahala |  |
| Mshiriki | | | |
| 3 |  | Jinsia |  |
| 4 |  | Umri |  |
| 5 |  | Hali ya ndoa |  |
| 6 |  | Nafasi/kazi |  |
| **SEHEMU II**  **TABIA ZA TIBA** | | | |
| 7 | Unadhani mkakati wa kuzuia Kifua Kikuu kwa wanaoishi na virusi vya Ukimwi ulianzia/umetoka wapi? | | |
| 8 | Je,hilo linaathiri vitu mtazamo wako/utekelezaji wake? | | |
| 9 | Unadhani makakati huu unahitaji mabadiliko? Mabadiliko yapi? | | |
| 10 | Ni aina gani ya taarifa au Ushahidi unaokuonyesha kuwa mkakati huu unafanya kazi? | | |
| 11 | Unadhani nin kinawavutia watumishi wengine kutekeleza mkakati huu? | | |
| 12 | Unamaoni gani juu ya ugumu wa mkakati huu? | | |
| 13 | Una vitendea kazi gani vinavyokusaidia kutekeleza mkakati huu? | | |
| 14 | Nini mtazamo wako juu ya vifungashio au taarifa zinazotumika kutekeleza mkakati huu? | | |
| 15 | Je, hiyo imaathiri vipi utekelzaji wa mkakati huo katika eneo lako? | | |
| **SEHEMU III**  **MAZINGIRA YA NDANI YA KITUO/ENEO LA UTEKELEZAJI** | | | |
| 16 | Unaelezea mahusiano yako na watumishi wengine na viongozi wako nje na ndani ya mazingira ya kazi? | | |
| 17 | Una maoni gani juu ya uhusiano wa taasisi/kito chako na wadau wemngine wenye ushawishi katika Makati huu? | | |
| 18 | Je mnakutana mara kwa mara na watumishi wenzio? | | |
| 19 | Je, ni kwa namna gani huwa unafahamu uwepo na mkakati/tiba au mfanayakazi mpya? Ulifahamu vipi kuhusu Tiba hii ya kuzuia Kifua Kikuu kwa watu wenye virusi vya Ukimwi? | | |
| 20 | Unazungumziaje utamadani wa taasisi yako au kitengo chako? | | |
| 21 | Unadhani utamaduni huo (Imani,mitazamo) unaathiri vipi utekelezaji wa wa mkakati huu? | | |
| 22 | Ni kwa kiasi gani mawazo mapya hupokelewa na kutumiwa kuongeza ufanisi wa utekelezaji wa mkakati huu? | | |
| 23 | Nini maoni yako juu ya mtazamo wa taasisi yako kwenye kutekeleza mkakati huu? | | |
| 24 | Watu wanahisi vipi juu ya mpango/utekelezaji /mchakato uliopo sasa katika kutekeleza mkakati huu? | | |
| 25 | Ni kwa namna gani utekelzaji wa mkakati huu unaendana na utamaduni na miiko ya taasisi au kituo chenu? | | |
| 26 | Ni kwa namna gani, utekelezaji wa mkakati huu unaendana unaendana na namna mnavyofanya kazi kwenye maeneo yenu? | | |
| 27 | Unaweza kuelezea ni kwa namna gani utekelezaji wa mradi huu umekuwa sehemu ya shughuli zenu za kila siku? | | |
| 28 | Unadhani ni kiasi gani uongozi wa taasisi yako umejihusisha na utekelezaji wa mkakati huu? | | |
| 29 | Je,mna rasilimali za kutosha kutekeleza mkakati huu? | | |
| 30 | Ni kwa namna gani mnapata rasilimali za kutekelza mkakati huu? | | |
| 31 | Binafsi na wenzio mna mafunzo gani katika kutekelza mkakati huu? | | |
| 32 | Mna tumia taarifa zipi zilizopo katika kutekeleza mkakati huu? | | |
| 33 | Huwa unamuuliza nani kama una swali juu ya utekelezaji wa mkakati huu? | | |
| **SEHEMU IV**  **MAMBO NJE YA TASISI/ENEO LA UTEKELEZAJI** | | | |
| 34 | Kwa kiasi gani taasisi yako ina taarifa juu ya uhitaji wa Makati huu? | | |
| 35 | Kwa kiasi gani unadhani mkakati huu unakidhi malengo na matazamio ya wagonjwa? | | |
| 36 | Kuna vikwazo gani wateja wako wanapata katika kufikia huduma hii? | | |
| 37 | Unajuaje mitazamo ya wateja wako juu ya mkakati huu? | | |
| 38 | Hiyo imaathiri vipi maamuzi yao kuhusiano na huduma hii? | | |
| 39 | Ni kwa namna gani unashairikiana na watu wengi nje ya taasisi yako? | | |
| 40 | Ni aina ipi ya taarifa mnazobadilishana juu ya huduma hii au kazi zenu kwa ujumla? | | |
| 41 | Kwa kiasi gani taasisi yako inakutia moyo kushirikiana na wengine nje ya taasisi? | | |
| 42 | Unaweza kutwambia unachojua juu ya taasisi nyingine katika utekelzaji wa huduma hii au huduma nyingine? | | |
| 43 | Kwa kiasi gani huduma hii inatolewa na taasisi nyingine? | | |
| 44 | Kwa kiasi gani idara nyingine ndani ya taasisi yako inatekeleza mkakati huu? | | |
| 45 | Kwa kiasi gani utekelezaji wa mkakati huu una faida kwa taasisi yako kwenye eneo lenu? | | |
| 46 | Ni aina ipi ya sera za wilaya,mkoa taifa zinaathiri utekelezaji wa mkakati huu? | | |
| 47 | Ni aina ipi ya motisha ya kifedha au isiyo ya fedha inasukuma utekelzaji wa mkakati huu? | | |
| 48 | Ni kitu gani cha kijamii au kiuchumi kinatia hamasa kutoa/kutumia huduma hii? | | |
| **SEHEMUV**  **TABIA ZA WATEKELEZAJI** | | | |
| 49 | Twambie ufahamu wako juu ya mkakati wa kuzuia ugonjwa Kifua Kikuu kwa watu wenye virusi vya ukimwi na namna unavyotekelzwa. | | |
| 50 | Wewe unahusika kwenye hatua ipi ya utekelezaji? | | |
| 51 | Unajisikiaje juu ya ufanisi ya huduma hiyo na utekelezwaji wake kwenye kituo chako? | | |
| 52 | Ni kwa kiwango gani unaweza kutoa/kutumia huduma hii? | | |
| 53 | Ni kwa kiasi gani unadhani wenzio wanaamini unaweza kutoa/kutumia huduma hii? | | |
| 54 | Ni kwa namna gani utambulisho wako kwenye taasisi unaathiri utekelezaji wa huduma hii? | | |
| 55 | Ni kwa namna gani motisha,misingi,Uwezo na namna yako ya kujifunza vinaathiri utekelezaji wako wa huduma hii? | | |
| **SEHEMU VI**  **MCHAKATO WA KUTOA HUDUMA** | | | |
| 56 | Unahusishaje wadau wengine katika utekelezaji wa huduma hii kwenye elimu,mafunzo? | | |
| 57 | Mnatumiaje watu wenye ushawishi? | | |
| 58 | Unazungumziaje uwepo na utumiaji rasmi wa viongozi wa ndani walioteuliwa kusimamia utekelezaji wa huduma hii? | | |
| 59 | Kwa namna gani mnawatumia mabingwa kwenye kutekelza huduma hii? Watu wanaotazamaje hawa watu? | | |
| 60 | Unawataarifuje wateja wako juu ya huduma hii? Nini mtazamo wao? | | |
| 61 | Ni kwa kiasi gani huduma hii inatolewa kama ilivyopangwa? | | |
| 62 | Mnapima vipi ufanisi wa utoaji wa huduma hii? | | |
| 61 | Unapataje mrejesho juu ya huduma hii? (wateja, wenzio, wakubwa zako wa kazi, nje ya taasisi/kituo) | | |

***ASANTE KWA KUSHIRIKI KWENYE UTAFITI HUU***
